# Transdermal estrogen gel vs oral estrogen after hysteroscopy for intrauterine adhesion separation: A prospective randomized study

**DOI:** 10.3389/fendo.2023.1066210

**Published:** 2023-03-08

**Authors:** Tianjin Yi, Xiaofang Zhang, Vani Gupta, Li Li, Qian Zhong

**Affiliations:** ^1^ Department of Gynecology and Obstetrics, West China Second University Hospital of Sichuan University, Chengdu, China; ^2^ Key Laboratory of Birth Defects and Related Diseases of Women and Children, Sichuan University, Ministry of Education, Chengdu, China; ^3^ College of Osteopathic Medicine, New York Institute of Technology College of Osteopathic Medicine in Long Island, NY, United States

**Keywords:** intrauterine adhesion, estrogen, transdermal, menstruation, pregnancy, hysteroscopy, randomized controlled trial

## Abstract

**Background:**

This randomized controlled trial (RCT) aimed to compare two different routes of postoperative estrogen treatment on the improvements of menstruation, postoperative endometrial thickness, and fertility outcomes in patients with moderate to severe intrauterine adhesions (IUA).

**Methods:**

This study prospectively included 78 women (age: 25 to 45 years) with moderate to severe IUA who underwent hysteroscopic resection of adhesions between March 2019 and October 2020. The enrolled patients were randomized 1:1 into either the transdermal gel group (n = 39) or the estradiol valerate oral tablet group (n = 39) on the day of receiving hysteroscopy. Postoperative endometrial thickness, AFS (American Fertility Society) score, estrogen level, and the pattern and amount of menstruation were compared. Pregnancy information was actively collected during 1-year follow-up after the operation.

**Results:**

The postoperative endometrium thickness was improved in both groups, and both groups gained menstruation improvement rates of 67%. For patients who underwent second-look hysteroscopy (17 from the oral group and 19 from the transdermal group), the mean AFS score declined greater than 2 in both groups. For patients with postoperative pregnancy intention, the pregnancy rates at 1-year follow-up after the procedures were 40.5% and 28% in the transdermal group and oral group, respectively. Although no statistically significant difference was observed between the two groups, patients in the transdermal group had a tendency toward increased pregnancy rate.

**Conclusions:**

Transdermal administration of estrogen is equally efficacious as oral estrogen in postoperative treatment of IUA patients with a relatively safe profile. It is very likely to broaden its indication to the field of IUA.

**Trial Registration:**

http://www.chictr.org.cn/showproj.aspx?proj=37197, identifier ChiCTR1900022110.

## 1 Background

Intrauterine adhesion (IUA) was first reported in 1948 by Asherman and defined as “traumatic amenorrhea”, also known as Asherman’s syndrome after his name (1). Trauma to the basalis layer of the endometrium can result in amenorrhea, dysmenorrhea, abnormal uterine bleeding, infertility, recurrent miscarriage, or abnormal placentation. Most troubling of all, once the endometrium is damaged beyond repair, a woman will be permanently infertile (2). Currently, the incidence of IUA is rising due to the increased number of abortions as well as dilation & curettage (3). There is no effective treatment for severe IUA to restore fertility function and menstrual physiology. The subsequent adhesion rate after hysteroscopic transcervical resection of adhesion (TCRA) is up to 60% (4), and the success rate of pregnancy varies from 22.5% to 66.1% in patients with moderate to severe IUA (5–8).

Postoperative use of estrogen after IUA separation, with or without progesterone, can help reduce the probability of adhesion re-formation. The AAGL (American Association of Gynecologic Laparoscopists) Practice Guideline also recommends post-operative estrogen therapy for 2-3 cycles, but does not specify the optimal dose and route of oestrogen therapy (Grade B evidence) (9). The dose of estrogen for IUA prevention varies from estradiol 2 mg to 12 mg daily in clinical practice (2, 7). The extended indication and prolonged duration of administration have increased concerns about the risk of impaired liver function and venous thromboembolism (10). Recent data has suggested that a high level of hepatic exposure to estrone following oral estradiol administration leads to increased thrombin generation, whereas transdermal estrogen avoids the entero-hepatic circulation, and therefore may be safer with respect to thrombotic risk (11). However, there is limited evidence regarding the route of administration of estrogen: transdermally vs orally in postoperative IUA prevention. To address this, a randomized controlled trial (RCT) was performed to compare two different routes of postoperative estrogen treatment on the improvements of menstruation, postoperative endometrial thickness, and fertility outcomes in patients suffering from moderate to severe IUA.

## 2 Methods

### 2.1 Ethical approval

This single-blinded RCT was approved by the Chinese Ethics Committee of Registering Clinical Trials (Approval No. ChiECRCT-20190011), and the study was registered in the Chinese Clinical Trial Registry Center (Registration No. ChiCTR1900022110). The study was conducted according to the Quality Management Practice for Drug Clinical Trials [Good Clinical Practice (GCP)] and the guidelines of the 1975 Declaration of Helsinki for human experimentation. Informed consent was obtained from each participant.

### 2.2 Patients

This study recruited 78 women (age 25 to 45 years) who underwent hysteroscopic surgery for IUAs in a university affiliated hospital from March 2019 to October 2020. The inclusion criteria for participants were as follows: (i) 25 years ≤ age ≤ 45 years; (ii) moderate or severe IUA according to the AFS (American Fertility Society) IUA scoring system (12); (iii) liver function, kidney function, and coagulation parameters are within the normal range; (iv) normal electrocardiogram results; and (v) normal ovarian function. Patients were excluded if they met any of the following criteria: (i) history of previous TCRA; (ii) suffering from diseases of the female reproductive system (including a history of genital tuberculosis); (iii) history of thrombosis, or family history of thrombosis or high risk for thrombosis; (iv) suffering from breast disease including breast tumors and breast hyperplasia.

### 2.3 Randomization, intervention, and blinding

The enrolled patients were randomized (1:1) on the day of receiving their hysteroscopy, in which 39 were randomized to the transdermal estrogen gel group and 39 to the estradiol valerate tablet (oral) group. The random number list was generated by using SPSS software (Version 19.0; SPSS Inc, IL). The investigators were blinded to the treatment allotment. Based on the pharmacokinetics data provided by the manufacturer of each drug, the transdermal estrogen gel contains 0.06% 17-beta estradiol (Jianmin Group Pharmaceutical Co. Ltd, China), that is 1.5 mg estradiol in 2.5 g gel. We chose 10 g of estradiol gel (containing 6 mg of 17-beta estradiol) to compare with 6 mg of estradiol valerate. The treatment group received 5 g twice daily of transdermal estrogen gel, and the control group received oral treatment with 3mg twice daily of estradiol valerate (Progynova, Bayer). All subjects received hormone treatment for two cycles.

### 2.4 Hysteroscopy

All patients received TCRA by two experienced surgeons under general anesthesia. A 9-mm working element and a 4-mm 30˚ telescope (both from Olympus, Japan) connected with a bipolar electrode needle or loop were placed into the uterine cavity to separate the adhesions. The cutting power were set at 80 W. An automated hysteroscopic distension pump delivered 0.9 percent sodium chloride into the uterine cavity at a distension pressure of 100 mmHg. The AFS scores were recorded according to the IUA scoring system. After the procedure, sodium hyaluronate gel was applied through intrauterine injection into the uterine cavity and intrauterine balloon was placed for 7 days in all patients. A second-look hysteroscopy was scheduled at 8 weeks (± 1 week) after the initial hysteroscopy and performed in the same manner as the initial procedure. Unfortunately, some patients refused the second-look hysteroscopy due to the improvement of menstrual volume and the hope of preparing for pregnancy as soon as possible. Prior to follow-up hysteroscopy, all subjects underwent examination to rule out pregnancy. The AFS scores were also recorded by a researcher who was unaware of the treatment option during the second-look hysteroscopy.

### 2.5 The outcomes measurements

Single-layer endometrial thickness on the 20th day of menstruation was measured by ultrasound before and after operation. The pre- and post-operative endometrial thickness and the amount of endometrial growth in the two groups were compared. The changes in the pattern and amount of menstruation before and after surgery in both groups were also compared. Since serum E2 levels were related to hormone treatment effect, the E2 level before and 5 days after the operation was tested and compared. The first- and second-look AFS scores were compared to assess endometrium rehabilitation. Menstruation and postoperative endometrial thickness are the primary outcomes, and pregnancy rate is the secondary outcome of our study. Pregnancy information was actively collected during 1-year follow-up after the operation.

### 2.6 Statistical analysis

The Shapiro Wilk Normality test was used to verify the normality of the variables. Variables with normal distribution were presented as means ± SD, and those with non-normal distribution were presented as median (interquartile range). Unpaired t-tests (normal distribution) or non-parametric Mann-Whitney-Wilcoxon tests (non-normal distribution) were used to calculate *P* values. Statistical significance was defined as a *P* value of <0.05, and all statistical tests were conducted on a two-sided basis. Data analysis and visualization were carried out using SPSS software (version 19.0; SPSS Inc, IL) and Graph-Pad Prism (version 5.0; Graph-Pad Software, 188 La Jolla, CA, USA), respectively.

## 3 Results

### 3.1 The baseline characteristics of the study subjects

**Figure 1:**
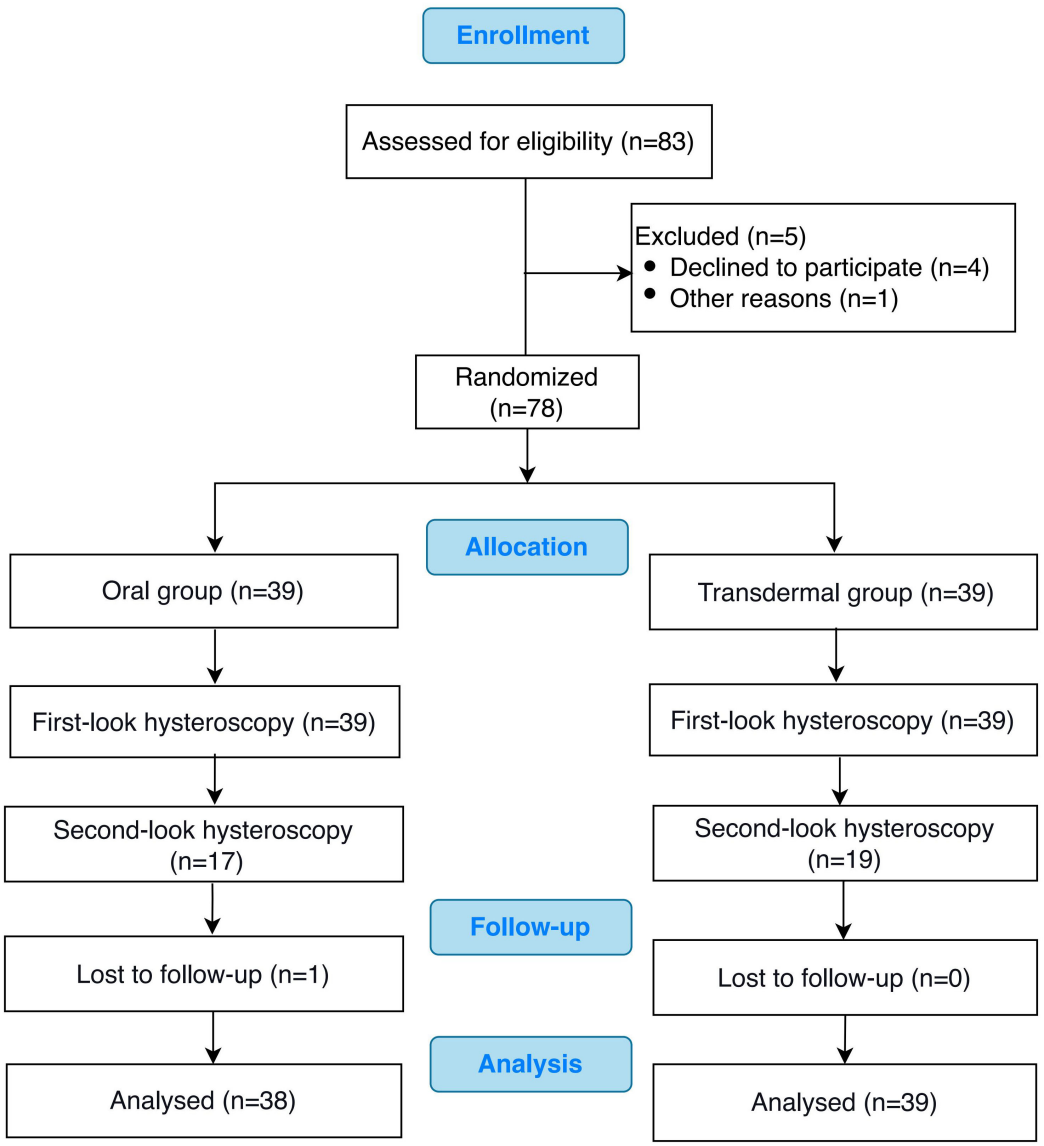
The CONSORT flow chart of the study.

The CONSORT flow chart of the participants in our study was presented in **Figure 1**. Of the 78 patients who met the inclusion criteria, 39 patients were randomized to the oral group and 39 to the transdermal group. The characteristics of the patients in both groups, including age, BMI, first-look AFS scores, pre-operative endometrial thickness, pre-operative serum estradiol (E2) level and pre-operative menstruation patterns were comparable (*P >*0.05) (**Table 1**).

**Table 1:** The comparison of baseline characteristics between the transdermal and oral groups.

	Oral group	Transdermel group	P value
Age (years) ^a^	31.26 ± 4.31	31.16 ± 4.51	0.92
BMI (kg/m^2^)^c^	20.83	20.83	0.85
Number of pregnancies^b^			0.21
< 2 (%)	8 (20.50)	4 (10.30)	
≥ 2 (%)	31 (79.50)	35 (89.70)	
Number of miscarriages^b^			0.60
< 2 (%)	9 (23.10)	11 (28.20)	
≥ 2 (%)	30 (76.90)	28 (71.80)	
Number of D&C relating to pregnancy^b^			0.31
< 2 (%)	9 (23.10)	13 (33.30)	
≥ 2 (%)	30 (76.90)	26 (66.70)	
AFS score^a^	8.08 ± 1.75	7.74 ± 1.74	0.40
Single-layer endometrial thickness (cm)^c^	0.25	0.2	0.98
Serum estradiol level (pg/ml)^c^	68.6	84.15	0.92
Menstrual pattern^b^			0.31
Abnormal period (%)	36 (92.3)	38 (97.4)	
Normal period (%)	3 (7.70)	1 (2.60)	

^a^Mean ± SD, comparison by using independent-samples t-test.

^b^Number (percentage), comparison by using Mann‐Whitney U test, a nonparametric test for independent sample t-test.

^c^Median, comparison by using the Mann‐Whitney U test.

### 3.2 The effects of the hormonal treatment

#### 3.2.1 Serum E2 levels

**Figure 2:**
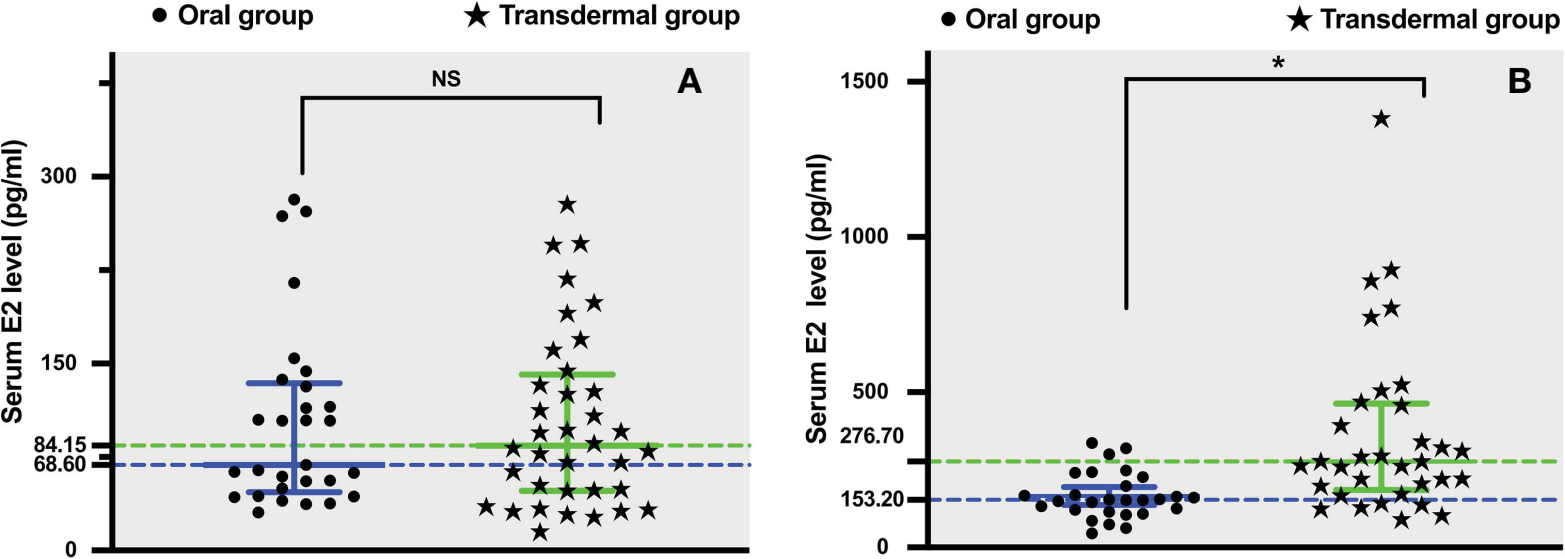
The comparison of serum estradiol levels between transdermal and oral groups. **(A)** Preoperative serum estradiol levels. **(B)** Postoperative serum estradiol levels. Results are presented as median, 25th and 75th percentiles of the distribution. Statistical analysis was performed using the Mann‐Whitney U test, a nonparametric test was used for independent sample t test. *P <0.05.

As pre- and post-operative E2 levels did not conform to normal distribution, the median value was used for comparative analysis. The pre-operative serum E2 level was 84.15 pg/ml (range: 15-278 pg/ml) and 68.6 pg/ml (range: 31-282 pg/ml) in the transdermal and oral group, respectively. There was no statistically significant difference between the two groups in terms of baseline serum E2 levels (*P*=0.916) (**Figure 2A**). At postoperative Day 5, the median serum E2 level of the transdermal group was 276.7 pg/ml (range: 90-1382 pg/ml), which was significantly higher than that of the oral group (median: 153.2 pg/ml, range: 45-336 pg/ml) (Wilcoxon rank sum *P* =0.000) (**Figure 2B**).

#### 3.2.2 AFS scores

**Figure 3:**
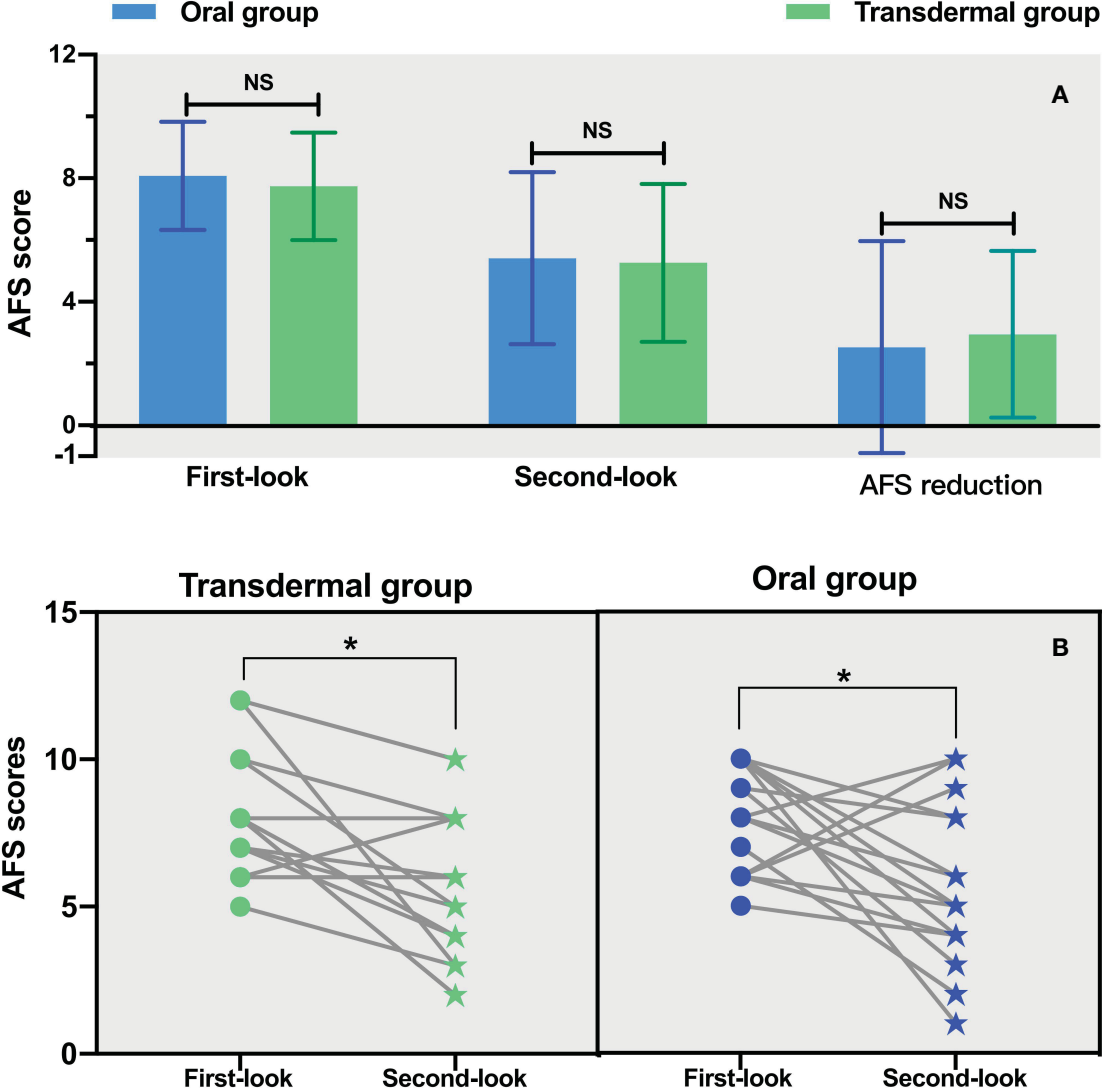
The comparison of AFS in both groups. **(A)** The comparison of the first-, second-look AFS as well as the AFS reduction. **(B)** The comparison of AFS between the first- and second-look hysteroscopy. Statistical analysis was performed using the paired t test. *P <0.05.

There was no significant difference in the first-look AFS scores between the two groups (8.08 ± 1.75 in the oral group and 7.74 ± 1.74 in the transdermal group, *P* =0.402) (**Figure 3A**). According to the AFS IUA scoring system, the severity of IUA in both groups was moderate to severe. Besides, there was no significant difference in the second-look AFS scores (*P* =0.868) as well as the AFS score reduction between the two groups (*P* =0.685) (**Figure 3A**). A second-look hysteroscopy after TCRA (within 2 months) was performed in 17 patients (43.58%, 17/39) of the oral group and 19 patients (48.72%, 19/39) of the transdermal group. The results showed that the second-look AFS scores were significantly improved in both groups (oral group: 8.08 ± 1.75 vs. 5.41 ± 2.79, *P* = 0.008; transdermal group: 7.74 ± 1.74 vs. 5.26 ± 2.56, *P* =0.0001) (**Figure 3B**).

### 3.3 The outcomes

#### 3.3.1 Endometrial thickness

**Figure 4:**
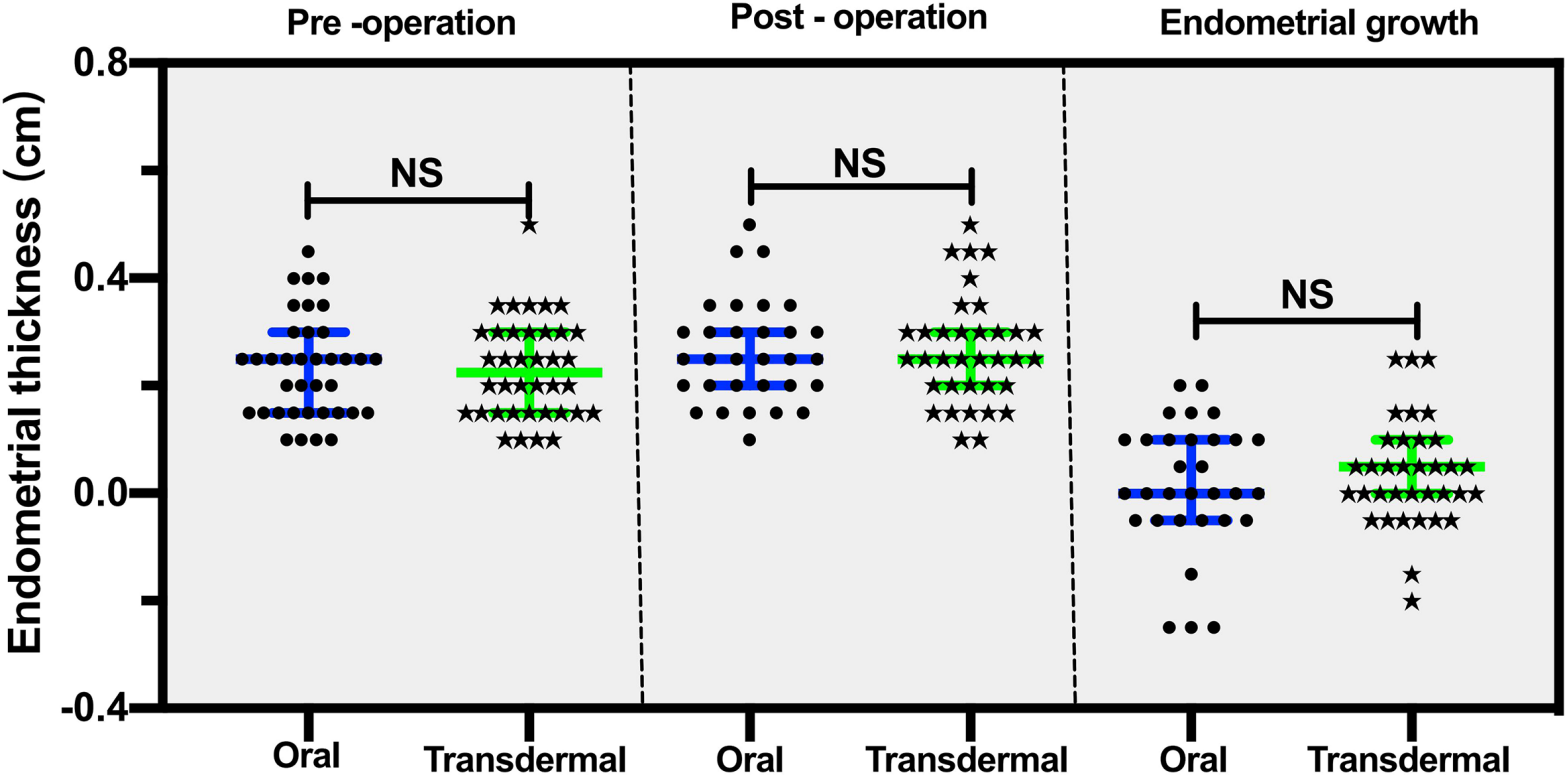
The comparison of pre- and post-operative endometrial thicknesses between the transdermal and oral group. The data are presented as median, 25th and 75th percentiles of the distribution. Statistical analysis was performed using the Mann‐Whitney U test, a nonparametric test was used for independent sample t test. NS, no significant difference.

The endometrial thickness before TCRA was comparable between the two groups (median: 0.2 cm in the transdermal group vs. 0.25 cm in the oral group, *P* =0.98) (**Table 1**; **Figure 4**). Similar endometrial growth after one-cycle of hormonal treatment in the two groups was observed (*P* =0.629) (**Figure 4**).

#### 3.3.2 Menstruation

**Figure 5:**
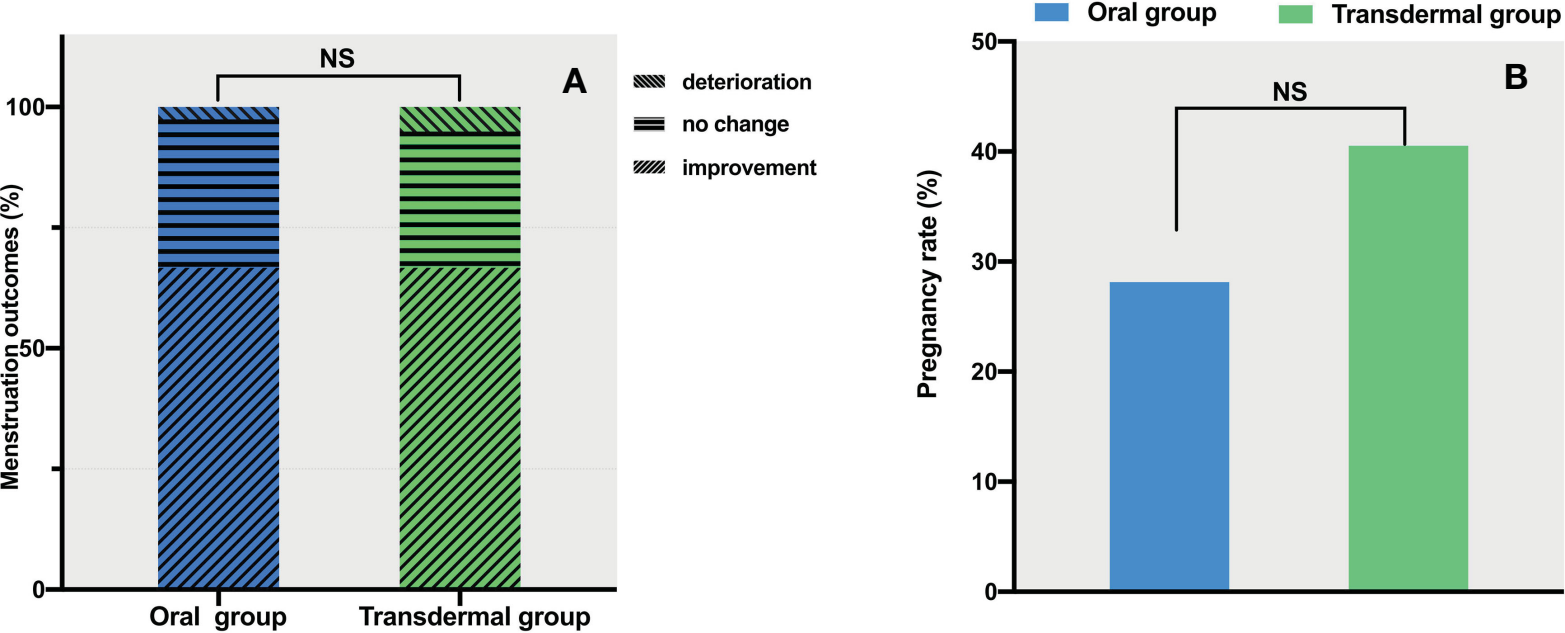
The comparison of menstrual and pregnant outcomes between the two groups. **(A)** Menstrual improvement outcomes. **(B)** Pregnancy rates. The data are presented as number (percentage), statistical analysis was performed using contingency table analysis. NS, no significance.

Hypomenorrhea is the most frequent complaint for IUA, and the menstrual pattern may reflect the amount of endometrium available for regeneration following TCRA. Before the procedures, 36 (92.3%) patients in the oral group and 38 (97.4%) patients in the transdermal group had pronounced hypomenorrhea. On the 2^nd^ month of follow-up, 67% of the patients in both groups showed increased menstruation. There were no significant differences in the proportion of increased (oral vs. transdermal: 26/39 vs. 26/39), decreased (oral vs. transdermal: 12/39 vs. 11/39) and unchanged (oral vs. transdermal: 1/39 vs. 2/39) menstruation (*P* = 1.0) (**Figure 5A**). After excluding those with normal menstruation, a separate comparison showed that there was no significant difference between the two groups in menstuation outcomes (*P* = 1.0) (**Table 2**).

**Table 2:** The comparison of menstruation outcomes in patients presented with pronounced hypomenorrhea.

Menstruation Outcomes	Oral	Transdermal	P
	n (%)	n (%)	
Improved	24 (66.7)	25 (65.8)	1
Unchanged	11 (30.6)	11 (28.9)	
Decreased	1 (2.7)	2 (5.3)	

Statistical analysis was performed by the Chi-Square Test.

#### 3.3.3 Pregnancy

In the oral group, the number of cases of intrauterine pregnancy, non-pregnancy, without pregnancy intention, and loss of follow-up were 9 (23%), 23 (59%), 6 (15%) and 1 (3%), respectively. In the transdermal group, the number of cases of intrauterine pregnancy, non-pregnancy, without pregnancy intention, and loss of follow-up were 15 (38.5%), 22 (56.4%), 2 (5%) and 0, respectively. After excluding the cases without pregnancy intention and loss of follow-up, there was no significant difference in pregnancy rates between the two groups (28% in the oral group vs. 40.5% in the transdermal group, *P* =0.28) (**Figure 5B**).

### 3.4 Adverse effect

During follow-up, both groups had few cases of reported mild symptoms like mastodynia. Two patients had moderate allergic reactions to the transdermal gel and were relieved with Loratadine. There was no thrombosis reported in either of the two groups. The liver function of all patients was normal during medication.

## 4 Discussion

An increasing amount of evidence has proven that postoperative estrogen therapy for IUA patients can help repair the endometrium, improve menstrual volume, and prevent the occurrence of re-adhesion to a certain extent (13–15). Currently, hormone therapy is initiated right after TCRA, but there is no consensus on an optimal dose, route, and duration of estrogen administration. Therefore, studies are required to determine the most favorable therapeutic schemes, including dose, route, and duration of estrogen therapy. Currently, the majority of clinicians prefer to use oral estrogen. However, high doses and prolonged estrogen use may increase concerns about complications, such as thrombosis and digestive side effects. Transdermal medication administration has grown in popularity due to its numerous advantages, for example, avoiding the liver’s first-pass metabolism, reducing the risk of damage to the gastrointestinal system *via* the oral route, increasing the chance of consistent patient use, and allowing drug administration at a constant steady interval. Previous studies have also demonstrated that patients with transdermal estrogen gel therapy had a lower thrombotic risk than those received oral estrogen therapy (16, 17). Estradiol gel can be a good candidate for estrogen supplement, which has been approved to be used for the treatment of moderate to severe vasomotor symptoms and vulvovaginal atrophy caused by menopause (18). Before embryo-implantation, it has been reported that transdermal administration of estrogen has a superior effect on endometrial preparation than other administration routes (19).

In this study, we evaluated the efficacy of two estrogen administration routes in different aspects. Both groups gained menstruation improvement rates of 67%, which suggest that postoperative transdermal administration of estrogen could achieve a comparable and satisfactory outcome of the endometrium function recovery as the oral estrogen. As for the recovery of the morphology of the endometrial cavity, both groups gained a mean AFS score reduction greater than 2, resulting in a favorable outcome in preventing re-formation of adhesion. For patients with postoperative pregnancy intention, the pregnancy rates after the procedures were 40.5% and 28% in the transdermal group and oral group, respectively. Although no statistically significant difference was observed between the two groups, patients in the transdermal group tended to have a higher pregnancy rate.

In our study, both oral and transdermal estrogen supplementation achieved, to some extent, elevated serum E2 level, endometrium growth, prevention of IUA re-formation and improved menstruation. However, the pregnancy outcome at 1-year follow-up seemed not so optimistic and the menstruation recovery did not correlate with the pregnancy rate. It has been reported that pregnancy after TCRA is also related to endometrial metabolism and angiogenesis, and even high level of serum E2 combined with anti-adhesion measures is hard to reverse the altered biochemical or vascular environment of the endometrium (20). Therefore, restoring reproductive function after surgery is more difficult than resuming menstruation. The estrogen supplementation could only serve as an auxiliary treatment for IUA, and the efficacy of the treatment may largely depend on the severity of IUA. There are a number of other unmeasured confounders among non-pregnant participants that could contribute to this low rate. Such conditions include, but not limited to: uterine/tubal/peritoneal diseases or endometriosis, male subfertility, sexual dysfunction, diminished ovarian reserve, etc. A thorough evaluation may be initiated 12 months after the TCRA in women who do not achieve a successful pregnancy to identify the reason.

The dosage of estrogen administration for IUA is heterogeneous in the published literatures, ranging from 2mg to 8mg (9). Our study used a compromised dosage of 6mg estrogen in the transdermal and oral group. From our study, both groups gained comparable and satisfactory outcome, i.e. 67% of menstruation improvement rates. However, our study does not aim to compare different dosages of estrogen but designed to compare the different routes of administration. So based on the design and results of our study, it’s hard to conclude what the optimal dosage for transdermal and oral estrogen treatment is. But we could suggest that it is safe and effective to use 6mg of estrogen transdermally or orally for postoperative administration of moderate to severe IUA. As far as we are aware, this is the only RCT comparing the efficacy of different routes of postoperative administration of estrogen: transdermally versus orally, in the management of moderate to severe IUA. This study provides a new option for postoperative estrogen treatment, which avoids liver damage, reduces the risk of thrombogenesis, and has the same therapeutic effect as oral estrogen in treating IUA.

Certain limitations must be considered when interpreting study findings. First, we missed the postoperative live birth outcome. Second, the number of patients with second-look hysteroscopy was small, and the overall sample size was relatively small as well. Lastly, the optimal dose of estrogen administration has not yet been determined. Given the prevalence of postoperative intrauterine adhesions, this is an area in need of well-designed trials to address questions regarding the optimal dosage of postoperative estrogen administration for IUA. Additionally, as the subsequent pregnancy after IUA treatment is at risk of miscarriages, our next step would be to investigate whether estrogen use improves long-term pregnancy outcomes, such as live birth rates.

## 5 Conclusions

Despite the limitations, our study shows that transdermal estrogen (gel) is equally efficacious as oral estrogen for postoperative treatment of IUA patients with a relatively safe profile. It is very likely to broaden its indication to the field of IUA.

## Data availability statement

The original contributions presented in the study are included in the article/supplementary material. Further inquiries can be directed to the corresponding author.

## Ethics statement

The studies involving human participants were reviewed and approved by China Ethics Committee of Registering Clinical Trials. The patients/participants provided their written informed consent to participate in this study.

## Author contributions

TY drafted the manuscript. XZ conducted literature search and collected the clinical data. VG polished the paper in English. LL conducted statistical analysis and interpreted results. QZ conceptualized the study and edited the manuscript. All authors contributed to the article and approved the submitted version.
